# Protocol for high-throughput genome-wide translocation sequencing-based profiling of T-cell receptor *α* and *β* gene rearrangements in mouse T cells

**DOI:** 10.1016/j.xpro.2026.104782

**Published:** 2026-08-13

**Authors:** Rui Luo, Yawei Song, Dingpeng Yang, Yutong Xie, Xiaoling Qian, Wei Wu, Hai-Qiang Dai

**Affiliations:** 1 First Affiliated Hospital of Chongqing Medical University, Chongqing Medical University, Chongqing 400016, China; 2 State Key Laboratory of Epigenetic Regulation and Intervention, Shanghai Institute of Biochemistry and Cell Biology, Center for Excellence in Molecular Cell Science, Chinese Academy of Sciences, University of Chinese Academy of Sciences, Shanghai 200031, China; 3 CAS Key Laboratory of Multi-Cell Systems, Shanghai Institute of Biochemistry and Cell Biology, Center for Excellence in Molecular Cell Science, Chinese Academy of Sciences, University of Chinese Academy of Sciences, Shanghai 200031, China

**Keywords:** sequence analysis, sequencing, immunology

## Abstract

Current T cell receptor (TCR) sequencing approaches have inherent technical limitations, and low-primer-bias techniques for quantitative genomic-level repertoire analysis remain limited. Here, we present high-throughput genome-wide translocation sequencing-based TCR sequencing (HTGTS-TCR-seq) as a genomic DNA-based protocol for low-bias analysis of TCR diversity. We describe steps for DNA fragmentation, LAM-PCR enrichment, streptavidin bead capture, adapter ligation, and PCR for library construction. This protocol enables quantitative profiling of TCR rearrangement products at the genomic DNA level.

For complete details on the use and execution of this protocol, please refer to Luo et al.^1^

## Highlights

• Low-bias genomic DNA-based protocol for analysis of TCR V(D)J rearrangements
• Design principles for bait primers targeting TCR *α* and *β* gene loci
• Guidance on sequencing depth and analysis of TCR rearrangement repertoires

## Abstract (editor highlights)

Publisher’s note: Undertaking any experimental protocol requires adherence to local institutional guidelines for laboratory safety and ethics.

## Graphical abstract

**Figure:**
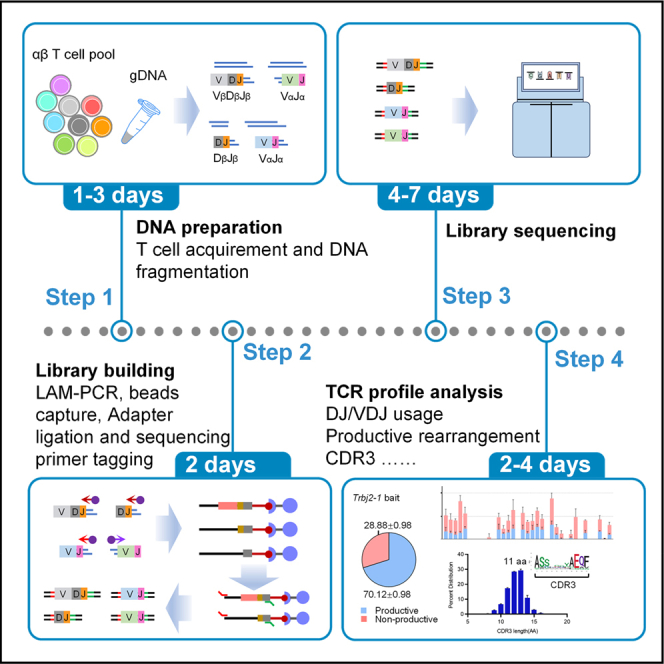

## Before you begin

T cell receptor (TCR) diversity is generated through V(D)J recombination during thymocyte development. In αβ T cells, the functional *Tcra* and *Tcrb* genes are generated from rearrangement of germline variable (V), diversity (D), and joining (J) segments. The *Tcrb* locus undergoes Dβ-to-Jβ before Vβ-to-DβJβ rearrangement at the double-negative (DN) stage. Productive TCRβ expression supports β-selection and promotes differentiation into CD4^+^CD8^+^ double-positive (DP) thymocytes.^2^ At the DP stage, the *Tcra* locus undergoes multiple successive Vα-to-Jα rearrangements until a functional *Tcra* gene is generated.^3^ These recombination events are initiated by the RAG1/2 recombinase complex at recombination signal sequences (RSSs) flanking V, D, and J gene segments.^4^ During this process, junctional insertions and deletions are generated at highly diverse complementarity-determining region 3 (CDR3) sequences, which are essential for antigen recognition. Therefore, characterizing TCR rearrangement products is important for studying T cell development, repertoire formation, aging, and immune dysfunction.^5^ However, commonly used approaches such as multiplex PCR and 5′RACE have inherent limitations, including primer bias, dependence on RNA quality, and incomplete capture of productive and nonproductive rearrangements at the genomic level.^6^^,^^7^

**Figure 1:**
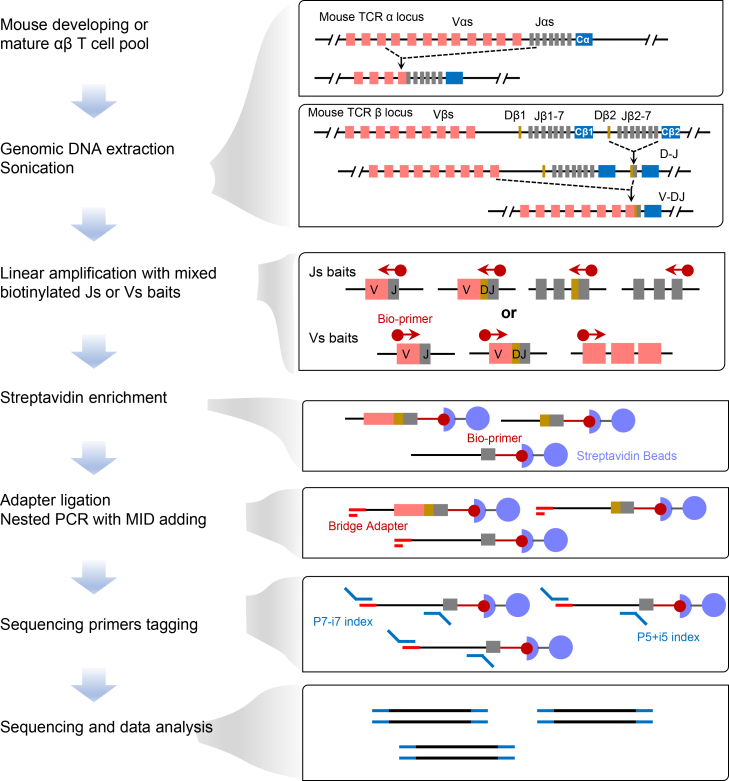
Overview and flow-chart of this protocol Genomic DNA (gDNA) from T-cell populations is sonicated and linearly amplified using mixed biotinylated primers, which anneal downstream of specific J or V-segments. The resulting biotin-labeled single-stranded DNA products are captured using streptavidin beads, ligated at the 3′ ends to a bridge adapter. Libraries are sequenced on Illumina NovaSeq X plus platforms with 150 bp paired-end reads. Sequence reads are processed and analyzed as described in Methods.

Here we present High-Throughput Genome-Wide Translocation Sequencing–based TCR sequencing (HTGTS-TCR-seq), a genomic DNA–based workflow designed for profiling *Tcra* and *Tcrb* rearrangements with reduced primer bias. This approach uses a limited set of J- or V-region biotinylated bio-primers to enrich recombination products, followed by adapter ligation, indexed PCR, library construction, and data analysis. HTGTS-TCR-seq enables analysis of segment usage, rearrangement efficiency, nonproductive rearrangements, and CDR3 diversity of productive rearrangement products (Figure 1). Accordingly, the method is particularly suited to applications focused on genomic DNA–level rearrangement products and repertoire features, rather than transcript-derived clonotypes alone (Table 1). Thus, this protocol provides a distinct genomic DNA-based approach for studying TCR recombination biology, especially in contexts where genomic rearrangement products are of primary interest. The protocol below is optimized for primary mouse T cells including thymocytes and peripheral T cells, but it has also been used for human T cells. It can be adapted according to genome assembly, locus, bait design, and biological question of interest.

**Table 1:** Comparison of representative methods for TCR repertoire analysis

Feature	HTGTS-TCR-seq	Multiplex PCR^8^^,^^9^^,^^10^	5′RACE^6^^,^^11^^,^^12^^,^^13^	Single-cell TCR-seq^14^^,^^15^
Representative method	This work	Representative bulk DNA-based multiplex PCR methods	Representative bulk RNA-based 5′RACE methods	Single-cell immune profiling methods and platforms (e.g., commercial 10× Genomics scTCR)
Input requirements	Genomic DNA	Genomic DNA	RNA	Single-cell RNA/cDNA
TCR profile	Alpha (α) & Beta (β) (independent or same reactions)	Beta (β)	Transcript-derived TCRα and TCRβ repertoires	Paired TCRα/β transcripts at single-cell resolution
Resolution	Population-level (bulk)	Population-level (bulk)	Population-level (bulk)	Single-cell
Clonal coverage	Moderate to broad, depending on bait design and DNA input	Variable, depending on primer-set composition	Broad for expressed transcript-derived repertoires	Broad for expressed clonotypes
Applications	Genomic DNA-level TCR rearrangement analysis	genomic detection of selected rearrangements	Bulk transcript-based repertoire profiling	Linking paired TCR clonotypes to transcriptional states, protein expression, and other single-cell features
Relative cost	Moderate	Low to moderate	Moderate	High
Major limitations	Requires relatively large DNA input; primer design constraints; no α/β pairing	Primer bias; limited locus coverage; no α/β pairing	RNA quality dependence; transcript abundance bias; no α/β pairing	Transcript-based only; higher workflow complexity and cost

### Primer design

**Timing: Variable**

**Figure 2:**
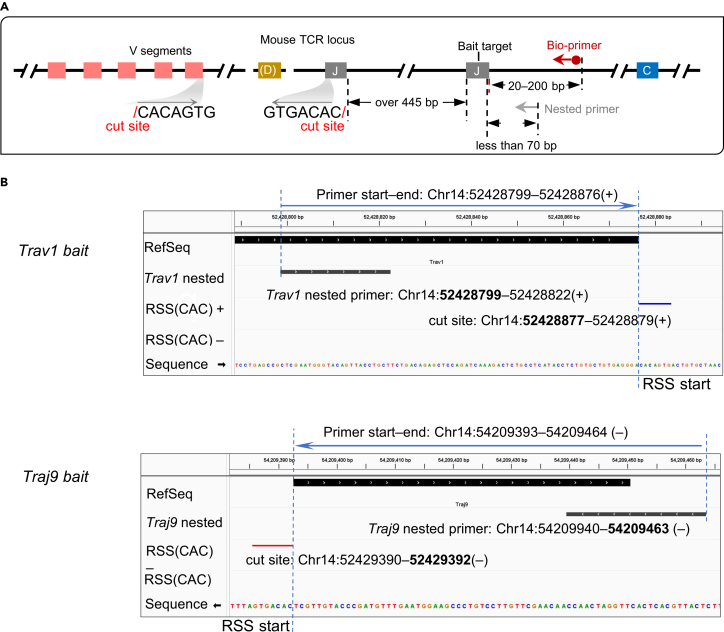
Schematic illustrating the principle of primer design (A) Schematic of primer design using a J-segment target as an example. (B) Examples of “START”, “END”, and “STRAND” parameters illustrated using *Trav1* bait (top) and *Traj9* bait (bottom) shown in IGV.

Two sets of primers, biotinylated bait primers and nested primers, are used to enrich TCR rearrangement products. A schematic example of the primer design strategy is shown in Figure 2. Primer positions are defined relative to the RSS cleavage site, and the upstream/downstream orientation should be adjusted according to the strand of the target segment. The bio-primer is used to capture target rearrangements over a relatively long distance. It should be designed within 20–200 bp downstream of the target sequence in the direction of primer extension. Thus, for targets located on opposite strands, such as J and V segments, the genomic position of the primer is adjusted according to strand orientation while maintaining the same functional relationship to the RSS cleavage site. Nested primers are used for specific amplification of the region of interest, and are positioned between the target segment and the bio-primer, at a distance of <70 bp from the target sequence. It could partly overlap the target segments while avoiding dimer and hairpin formation. Primers should be designed with annealing temperatures of 59°C–60°C and GC contents of 40%–50%, preferably not exceeding 60%. Baits can be selected from either functional or pseudogene segments that have been validated to undergo rearrangement by 5′RACE or multiplex PCR. To minimize overlap and interference among primers, bait primers, including the bio-primer and nested primer, should not be placed in close proximity to one another. When mixed primer sets are used, primer interference and capture efficiency should be evaluated both individually and in combination.

For the densely organized mouse Jβ regions, we initially designed bio-primers for Jβ1-1, Jβ1-3, Jβ1-4, Jβ2-1, and Jβ2-7, with bio-primers spaced at least 445 bp apart. Based on performance testing, Jβ1-1, Jβ2-1, and Jβ2-7 were selected as the final primer set, representing different regions of the locus, with bio-primers spaced over 1000 bp apart. For the *Tcra* locus, bio-primers were designed at frequently used Jα or Vα segments spanning proximal to distal regions, avoiding highly similar or repetitive segments, guided by published 5′RACE or quantitative PCR datasets. Baits for pseudogene segments involved in rearrangements were also included to comprehensively improve coverage of rearrangement products.

### Innovation

This protocol provides a practical workflow for genomic DNA-based analysis of TCRα and TCRβ rearrangement products. Compared with conventional multiplex PCR-based approaches, it uses a limited set of biotinylated bait primers together with linear amplification, thereby reducing reliance on large multiplexed primer pools. Compared with RNA-based methods, it can recover rearrangement information directly from genomic DNA, including certain pseudogene-associated events that may not be represented in transcript-derived datasets. The workflow is described for primary mouse T cells but can be adapted to human samples.^1^ It supports analysis of segment usage, productive and nonproductive rearrangements, rearrangement status, and CDR3 features. Together, this protocol adapts the HTGTS strategy into a practical, reduced-bias workflow that integrates library preparation, downstream analysis, and troubleshooting guidance for TCR repertoire profiling.

### Institutional permissions

All animal experiments were approved by the Institutional Animal Care and Use Committee of the Institute of Biochemistry and Cell Biology, Center for Excellence in Molecular Cell Science, Chinese Academy of Sciences, under protocol number SIBCB-S648-2110-034.

## Key resources table

**Table:** 

REAGENT or RESOURCE	SOURCE	IDENTIFIER
**Critical commercial assays**		

PBS (1×)	Dalian Meilun	MA0015-2
Fetal Bovine Serum	EXCELL	FSP500
Tris	ABCONE	T15033
sodium chloride	hushi	10019318
Sodium dodecyl sulfate	hushi	30166428
Ethylenediamine tetraacetic acid disodium salt dihydrate	hushi	10009717
Triton™ X-100	Sigma-Aldrich	T8787-50ML
Proteinase K	lifefeng	RE111-02
Isopropanol	hushi	80109218
Ethanol absolute	hushi	10009218
VAHTS DNA Clean Beads	Vazyme	N411-02
DB MYONE STREPTAVIDIN C1	Thermofisher-Invitrogen	65002
Super pure dNTP Mixture (2.5 mM each)	TIANGEN	CD111-03
TransStart® FastPfu DNA Polymerase	TransGen Biotech	AP221-03
EasyTaq® DNA Polymerase	TransGen Biotech	AP111-02
T4 DNA Ligase	Thermo	EL0012
PEG8000	Thermo	EL0012
Ethidium bromide stock solution,10 mg/mL	sangon	A500328-0005
Agarose	Yeasen	10208ES60
100 bp DNA Ladder	TIANGEN	MD109-02
CD4/CD8 (TIL) MicroBeads, mouse	Miltenyi	130-116-480
LS Separation columns	Miltenyi	130-042-401
MS Separation columns	Miltenyi	130-042-201
Equalbit 1 × dsDNA HS Assay Kit	Vazyme	EQ121-02
DNeasy Blood & Tissue Kit	QIAGEN	69504
Universal DNA Purification and Recovery Kit	TIANGEN	DP214-03

**Antibodies**		

APC/Cyanine7 anti-mouse CD4 (dilute 1:400)	Biolegend	100414
PerCP/Cyanine5.5 anti-mouse CD8a (dilute 1:1000)	Biolegend	100734
APC anti-mouse CD8a (dilute 1:400)	Biolegend	100712
FITC anti-mouse TCR β chain (dilute 1:400)	Biolegend	109205
FITC anti-mouse/human CD44 (dilute 1:400)	Biolegend	103005
PE anti-mouse CD25 (dilute 1:1000)	Biolegend	102007
Fixable Viability Stain 510 (dilute 1:500)	BD Pharmingen	564406

**Experimental models: Organisms/strains**		

Mouse: C57BL/6: Wild-type (C57BL/6J), 4 to 6-weeks-old, male and/or female	Shanghai Lingchang Biotech Limited Company	B6

**Software and algorithms**		

FlowJo	BD	https://www.flowjo.com/
Prism 10	GraphPad Software	https://www.graphpad.com/scientific-software/prism/
cutadapt	Martin et al.^16^	https://cutadapt.readthedocs.io/en/stable/
seqtk	Shen et al.^17^	https://github.com/lh3/seqtk
fastq-join	Aronesty^18^	https://github.com/brwnj/fastq-join
fastx	FASTX-Toolkit: FASTQ/a short-reads pre-processing tools.	http://hannonlab.cshl.edu/fastx_toolkit/
IgBLAST	Ye et al.^19^	https://ncbi.github.io/igblast/

**Other**		

Nanodrop	Eppendorf	NDL
Bioruptor	Qsonica	Q800-R3
PCR thermocycler	TIANGEN	OSE-GP-06
Magnet rack	Stemcell	18000
Centrifuge	Eppendorf	Cat#5406000291
Qubit	Invitrogen	Qubit4
Electrophoresis system	Tanon	EPS300
0.5 mL BioruptorPlus Microtubes	diagenode	Cat#C30010013
1.5 mL Microtubes	Axygen	Cat#MCT-150-C
0.2 mL PCR strip tubes	Genebrick	Cat# GP020805

## Materials and equipment

**Table:** 2× Binding & Wash (BW) buffer

Reagent	Final concentration	Amount
NaCl (5 M)	2 M	20 mL
Tris-HCl (pH 7.4) (1 M)	10 mM	500 μL
EDTA (0.5 M)	1 mM	100 μL
ddH_2_O	N/A	29.4 mL
Total	N/A	50 mL

1× BW can be prepared by diluting 2× BW with an equal volume of ddH_2_O. This buffer can be stored at room temperature for up to 1 year.

**Table:** Cell lysis buffer

Reagent	Final concentration	Amount
NaCl (5 M)	200 mM	2 mL
Tris-HCl (pH 7.4) (1 M)	50 mM	2.5 mL
EDTA (0.5 M)	5 mM	500 μL
SDS (10%)	0.2% (wt/vol)	1 mL
ddH_2_O	N/A	44 mL
Total	N/A	50 mL

Add 1% proteinase K before use. The buffer can be stored at room temperature for up to 6 months.

**CRITICAL:** SDS is harmful and irritating to the skin, eyes, and respiratory tract. Wear gloves and a lab coat. Avoid inhalation and direct contact with skin or eyes.

**Table:** Dilute TE

Reagent	Final concentration	Amount
Tris-HCl (pH 8.0) (1 M)	10 mM	500 μL
EDTA (0.5 M)	0.1 mM	10 μL
ddH_2_O	N/A	49.49 mL
Total	N/A	50 mL

The buffer can be stored at room temperature for up to 1 year.

**Table:** Oligonucleotides

Primer	Sequence (5’→3′)
Bio-Jβ1-1	/5-Biotin/GAATATGGACACGGAGGACATGC
Bio-Jβ1-4	/5-Biotin/TGCCTTTGCAGTCCTCACCTTG
Bio-Jβ2-1	/5-Biotin/GGTCTTTGTGGCTGACTGTCCTAC
Bio-Jβ2-3	/5-Biotin/AACTGGCTCTGGCTATCCTTAGC
Bio-Jβ2-7	/5-Biotin/AACCCTGGTCTACTCCAAACTACTC
I5_Jβ1-1	ACTCTTTCCCTACACGACGCTCTTCCGATCT*CAAAAG*CCTACAACTGTGAGTCTGGTTCCTT
I5_Jβ1-4	ACTCTTTCCCTACACGACGCTCTTCCGATCT*CACTCA*TACATACCCAGGACAGACAGCTTG
I5_Jβ2-1	ACTCTTTCCCTACACGACGCTCTTCCGATCT*CATGGC*TACCTAGGACGGTGAGTCGTGTC
I5_Jβ2-3	ACTCTTTCCCTACACGACGCTCTTCCGATCT*CAAACA*ACTTACCGAGAACAGTCAGTCTGG
I5_Jβ2-7	ACTCTTTCCCTACACGACGCTCTTCCGATCT*CTAGCT*TACCTAAAACCGTGAGCCTGGT
Bio-Vα1	/5-Biotin/TACCAGCAACGTGAAGGCCAAG
Bio-Vα2	/5-Biotin/GGACTATGTGGTAAATGAAGTGGCAT
Bio-Vα17	/5-Biotin/CТCCTCAAATACATCACAGGAGACAC
Bio-Vα20	/5-Biotin/CACGCTCCTAATAGACATTCGCTC
I5_Vα1	ACTCTTTCCCTACACGACGCTCTTCCGATCT*CAAAAG*CTCGAATGGGTACAGTTACCTGCT
I5_Vα2	ACTCTTTCCCTACACGACGCTCTTCCGATCT*ATTCCT*TGACCGGAAGCTCAGCACTC
I5_Vα17	ACTCTTTCCCTACACGACGCTCTTCCGATCT*CAACTA*GAGTAACTCCTCTTTCAACCTGAAG
I5_Vα20	ACTCTTTCCCTACACGACGCTCTTCCGATCT*CACGAT*CGCGTCTCCTTACATATAACAGCTG
Bio-Jα61	/5-Biotin/ACTATTCCTGAAGGAACACTTCCATC
Bio-Jα56	/5-Biotin/AGCTCAGTTGTTCCTCACTGAAAC
Bio-Jα45	/5-Biotin/CCTGTCTACTTCTCATTTAGGCTCC
Bio-Jα18	/5-Biotin/CCCTGGAGTAGAAAGAAACCTACTC
Bio-Jα9	/5-Biotin/AGCCACAGGTAACTCCTTATCTCC
I5_Jα61	ACTCTTTCCCTACACGACGCTCTTCCGATCTINDEX*CTTGCTGAGTTTCATGAGTCTTCCAG*
I5_Jα56	ACTCTTTCCCTACACGACGCTCTTCCGATCTINDEX*CCTGGTATAACACTCAGAACGGTTC*
I5_Jα45	ACTCTTTCCCTACACGACGCTCTTCCGATCTINDEX*TTACAGGGCTGGATGATCAGCTG*
I5_Jα18	ACTCTTTCCCTACACGACGCTCTTCCGATCTINDEX*CTCACCAGGTATGACAATCAGCTG*
I5_Jα9	ACTCTTTCCCTACACGACGCTCTTCCGATCTINDEX*TCATTGCACTCACTTGGATCAACC*
BAup_10N	/Phor/CCACGCGTGCTCTACANNNTNNNNTNNNAGATCGGAAGAGCACACGTCTG AACTCCAGT-NH_2_
BAdown	TGTAGAGCACGCGTGGNNNNNN-NH_2_
I7-I13	CAGAAGACGGCATACGAGATINDEXGTGACTGGAGTTCAGACGTGTGC
P5-I5	AATGATACGGCGACCACCGAGATCTACACTCTTTCCCTACACGACGCTCTTCCGATCT
P7-tag	CAAGCAGAAGACGGCATACGAGAT

***Note:*** The ‘N’ in the bridge adapter indicates the random nucleotide. The italic characters in i5 primer illustrate the nested primer designed on the genome. The underlined “INDEX” sequence in i5 and i7 primers illustrates the index of each primer. These index sequences can be designed and synthesized according to the index of the Illumina platform.

## Step-by-step method details

### Primer preparation

**Timing: 1 h**

1. Prepare bio-primer and nested primer dilutions.
  a. Dilute each bio-primer in TE buffer to prepare a 100 μM stock solution.
  b. Dilute an aliquot of the 100 μM stock to prepare a 10 μM stock in TE buffer.
  c. Dilute an aliquot of the 10 μM stock in nuclease-free water to prepare a 1 μM working solution for use in experiments.
    ***Optional:*** Mix equal volumes of the 1 μM bio-primer solutions to generate a pooled primer mix for use after validating the performance of each primer.
  d. Dilute each nested primer in TE buffer to prepare a 100 μM stock solution.
  e. Dilute an aliquot of the 100 μM stock to prepare a 10 μM working stock in nuclease-free water. Primers should be stored at −20°C for up to 2 years.
2. Prepare bridge adapter dilutions.
  a. Dissolve the upper and lower oligonucleotides in TE buffer to prepare 400 μM stock solutions respectively.
  b. Mix the upper and lower oligonucleotides in equal volumes to generate an adapter mix with a final concentration of 200 μM.
  c. Boil the mixture for 5 min, then allow it to cool gradually to room temperature for annealing.
  d. Add three volumes of ddH_2_O to aliquot the adapter solution, and store it frozen. Avoid repeated freeze-thaw cycles. The annealed adapter should be stored at −20°C for up to 2 years.

### Sample preparation

**Timing: 6 h**

Prepare single-cell suspensions from lymphoid tissues and isolate the T cell populations of interest by direct processing or flow cytometric sorting.

3. Prepare single-cell suspensions from thymi or spleens of 4–6-week-old mice by mechanical dissociation and filtration through a 70-μm nylon cell strainer.
4. Lyse red blood cells if necessary, pellet the remaining cells by centrifugation, and resuspend the cells in PBS supplemented with 2% fetal bovine serum (FBS).
5. Isolate the cell population of interest directly or by flow cytometric sorting.
  ***Note:*** The T cell population and mouse age should be selected depending on the biological question of interest.
  a. For isolation of DN3 thymocytes from WT mice, first deplete CD4^+^ and CD8^+^ thymocytes using CD4/CD8 (TIL) MicroBeads according to the manufacturer’s instructions. Collect the flow-through fraction for subsequent antibody staining and cell sorting.
    ***Note:*** This enrichment step reduces the proportion of mature thymocytes and improves recovery of DN populations during sorting.
  b. For isolation of preselection DP thymocytes or splenic T cell populations, use the corresponding single-cell suspensions directly for antibody staining and flow cytometric sorting.
  c. For analysis of whole thymocytes, collect the unfractionated thymic single-cell suspension directly for downstream processing.
6. Stain cells with the indicated fluorochrome-conjugated antibodies at a 1:400 dilution, unless otherwise specified. Use Fixable Viability Stain 510 according to the manufacturer’s instructions.
7. To isolate DN3 thymocytes, stain the enriched WT thymocyte fraction with PE-anti-CD25, FITC-anti-CD44, and Fixable Viability Stain 510 after CD4/CD8 lineage dump. Define DN3 cells as CD4^-^CD8^-^CD44^-^CD25^+^ (Figure 3B).

**Figure 3:**
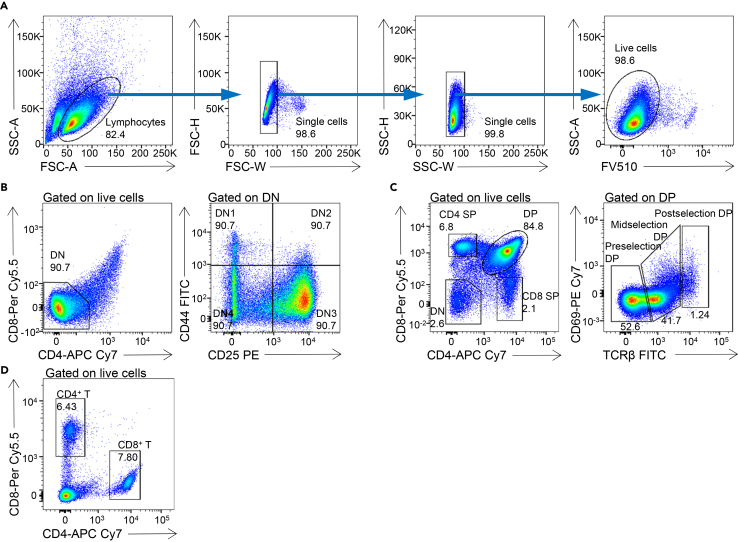
Representative flow cytometric gating strategy applied for cell sorting (A) Schematic gating strategy for live cells. (B) Schematic gating strategy for DN3 thymocytes after CD4/CD8 bead depletion. (C) Schematic gating strategy for preselection DP thymocytes. (D) Schematic gating strategy for splenic CD4^+^/CD8^+^ T cells.
8. To isolate preselection DP thymocytes from young mice, usually 4–6 weeks old, stain total thymocytes with APC-Cy7-anti-CD4, PerCP/Cyanine5.5-anti-CD8, PE-Cy7-anti-CD69, FITC-anti-TCRβ, and Fixable Viability Stain 510. Define preselection DP thymocytes as CD4^+^CD8^+^CD69^-^TCRβ^lo^ (Figure 3C).
9. To isolate splenic CD4^+^ or CD8^+^ T cells, stain splenic single-cell suspensions with APC-Cy7-anti-CD4, PerCP/Cyanine5.5-anti-CD8, and Fixable Viability Stain 510, and gate on live CD4^+^ or CD8^+^ lymphocytes as required (Figure 3D).
10. Sort the indicated populations using a FACSAria III or FACSAria Fusion cell sorter and collect the purified cells for downstream genomic DNA preparation.

**CRITICAL:** Keep cells on ice whenever possible and minimize the interval between staining and sorting to preserve viability and sample quality.

**Pause point:** Sorted cells can be pelleted and stored at −80°C before genomic DNA extraction, although freshly processed samples are preferred.

### Genomic DNA extraction

**Timing: 2 days**

Extract genomic DNA from isolated or sorted T cell populations.

11. T cells can be directly isolated or sorted from thymus, spleens, PBMCs, or in vitro cultures. Count the live cells.
  a. Centrifuge at 600 × *g* for 3 min, aspirate the supernatant.
  b. Wash with 1 mL PBS.
  c. Repeat step a and b once.
12. Resuspend 1 × 10^6^ to 1 × 10^7^ mouse T cells in 500 μL of cell lysis buffer and incubate at 56°C with shaking at 900 rpm overnight (10–16 h).
13. Add 1 mL of ethanol directly into the microtube, gently invert the microtube until the liquid is mixed thoroughly.
14. Centrifuge at 12,000 *g* for 5 min at 4°C.
15. Aspirate the supernatant and wash the pellet with 1 mL of 70% (vol/vol) ethanol.
16. Repeat steps 12 and 13 once.
17. Aspirate the supernatant completely, and dissolve the pellet in 110 μL of diluted TE buffer at 900 rpm, 56°C overnight (10–16 h).
18. Check the concentration with NanoDrop; the A260/280 value should be around 1.8.

***Optional:*** For samples containing 2 × 10^5^ to 1 × 10^6^ cells, a commercial genomic DNA extraction kit is recommended (e.g. DNeasy Blood & Tissue Kit, 69504, Qiagen). For samples containing fewer than 2 × 10^5^ cells, the resulting low DNA yield may compromise experimental performance. Kit-based extraction can also be used as an alternative to steps 9–15. DNA concentration should still be measured by NanoDrop, although the A260/280 ratio may be less reliable.

***Note:*** Genomic DNA can be stored at −20°C for months.

### Genome DNA fragmentation

**Timing: 1 h**

Sonicate genomic DNA to generate fragments of appropriate size for LAM-PCR.

19. Transfer 0.2–20 μg DNA to a BioruptorPlus Microtube and adjust the volume to 110 μL with nuclease-free water. Troubleshooting 1.

***Note:*** Although 0.2 μg DNA can also be used to construct a library, a relatively large amount of input DNA is recommended.

20. Pre-cool the Bioruptor to 4°C, then place the microtubes into the Diagenode Bioruptor adapter. Ensure balanced loading.
21. Set up the sonication conditions as follows: total duration, 1 min 30 s; 30 s on/30 s off cycles; 30% amplitude.

***Note:*** Temperature can be maintained at 4°C–6°C during sonication process.

**Figure 4:**
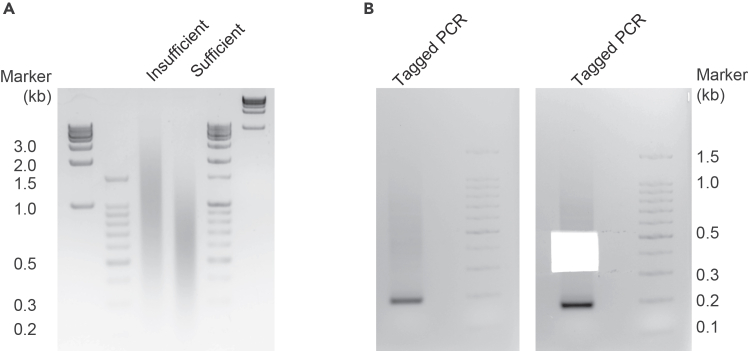
Representative smear of sonicated DNA and library size selection after tagged PCR (A) Properly sonicated genomic DNA fragments present as a smear ranging from 200 bp to 1.5 kb, with a peak distribution around 750 bp. (B) Tagged PCR products of 300–500 bp are excised and purified for subsequent sequencing.

***Note:*** If the target size is not achieved, another round of 30 s sonication is recommended. Check the sonication efficiency one more time to determine the band distribution.

22. Take 50–300 ng of sonicated DNA and run it on an agarose gel to assess the fragmentation efficiency. The DNA should be a smear ranging from 200 bp to 1.5 kb, with a peak around 750 bp (Figure 4A).

**Pause point:** Sonicated genomic DNA can be stored at −20°C for months. Avoid freeze-thaw.

### LAM-PCR

**Timing: 5 h**

Linearly amplify fragmented genomic DNA using biotinylated bait primers to enrich TCR rearrangement products.

23. Set up LAM-PCR reactions for each sample as below:

**Table:** LAM-PCR reaction master mix

Reagent	Amount (μL)
5× FastPfu buffer	40
dNTPs (2.5mM each)	4
Bio-primer (1 μM)	4
FastPfu	2
Sonicated DNA	100
Nuclease-free water	50

Divide each sample into four 50-μL reactions.

***Note:*** If mixed primers are used in a single library, dilute each primer stock to 1 μM with nuclease-free water and mix equal volumes of each primer before use.

24. Perform PCR using the following conditions:

**Table:** PCR cycling conditions

Steps	Temperature	Time	Cycles
Initial Denaturation	95°C	2 min	1
Denaturation	95°C	30 sec	80 cycles
Annealing	58°C	30 sec	
Extension	72°C	1 min 30 sec	
Final extension	72°C	2 min	1
Hold	10°C	forever

**CRITICAL:** Proceed directly to bead purification after LAM-PCR. Do not leave the reaction at 4°C for extended periods or subject it to freeze-thaw cycles before cleanup. Prolonged storage of the unpurified PCR mixture is not recommended, as residual biotinylated primer and other reaction components may compromise product recovery and reduce the efficiency of subsequent streptavidin-based enrichment.

### Bead purification

**Timing: 1 h**

Remove excess primers and reaction components using VAHTS DNA Clean Beads.

25. 30 min before the end of PCR, equilibrate VAHTS DNA Clean Beads from 4°C to room temperature.
26. Add an equal volume of DNA Clean Beads to the PCR product and mix thoroughly by pipetting. Incubate at room temperature for 5 min.
27. Place the PCR tube on a magnetic rack, and stand for 5 min at room temperature.
28. Remove the supernatant and wash the beads with 200 μL of fresh 80% ethanol. Incubate at room temperature for 30 s. Repeat the wash once.

***Note:*** Do not disturb the beads during incubation.

29. Remove the supernatant and air-dry the beads for 2–5 min.
30. Resuspend the beads in 35 μL of 10 mM Tris-HCl (pH 7.5), and mix thoroughly by pipetting up and down 15–20 times.
31. Incubate at room temperature for 5 min to elute the DNA.
32. Place the PCR tube on the magnetic rack. Stand for 5 min at room temperature, and transfer the supernatant to a new PCR tube.

**Pause point:** Purified DNA can be stored at −20°C for months. Avoid repeated freeze-thaw cycles.

### Streptavidin enrichment

**Timing: 4.5 h**

Capture biotinylated single-stranded LAM-PCR products using streptavidin beads for subsequent on-bead adaptor ligation.

33. Transfer 20 μL of Dynabeads MyONE C1 streptavidin beads into a new 1.5 mL tube.
34. Wash the beads with 600 μL of 1× BW buffer by pipetting.
35. Put the tube on a magnetic rack, incubate for 1 min, and remove the supernatant.
36. Repeat steps 16 and 17 twice.
37. Resuspend the beads with 20 μL of 10 mM Tris-HCl (pH 7.5).
38. Add the beads to the purified PCR product, and add 40 μL of 5 M NaCl and 2 μL of 0.5 M EDTA to the product, and mix thoroughly.
39. Incubate on a rotator at 8 rpm for 4 h at room temperature.

### On-bead adapter ligation

**Timing: 12–16 h**

Ligate the bridge adaptor to bead-bound DNA products to generate templates suitable for nested PCR amplification.

40. Put the tube on a magnetic rack, stand for 1 min, and remove the supernatant.
41. Wash the beads with 200 μL of 1× BW by pipetting.
42. Repeat steps 22–23 twice.
43. Resuspend the beads with 30.6 μL of 10 mM Tris-HCl (pH 7.5).
44. Set up on-bead adapter ligation reactions for each sample as below:

**Table:** 

Reagent	Amount (μL)
10× T4 ligase buffer	6
Bridge adapter (50 μM)	2.4
T4 ligase	3
50% PEG8000	18
DNA-beads complex	30.6

***Note:*** Do not spin the beads.

45. Set thermocycler incubation conditions as follows:

**Table:** PCR cycling conditions

Temperature	Time
25°C	1 h
22°C	2 h
16°C	Hold overnight

### Nested PCR

**Timing: 2 h**

Amplify adaptor-ligated DNA products with indexed primers to generate sequencing-ready libraries for downstream analysis.

46. Add 60 μL of 2× BW and 80 μL of 1× BW, mix by pipetting.
47. Place the tube on a magnetic rack and incubate at room temperature for 1 min to allow the beads to separate. Remove the supernatant.
48. Wash the beads with 200 μL of 1× BW by pipetting.
49. Repeat steps 29–30 twice.
50. Wash the beads with 200 μL of 10 mM Tris-HCl (pH 7.5) by pipetting.
51. Remove the supernatant and resuspend the beads with 50 μL of 10 mM Tris-HCl (pH 7.5).
52. Set up nested PCR reactions for each sample as below:

**Table:** 

Reagent	Amount (μL)
10× EasyTaq buffer	10
dNTPs (2.5 mM each)	8
I5 primer	4
I7 primer	4
EasyTaq	1
DNA-beads complex	50
Nuclease-free water	23

Divide each sample into two 50-μL reactions.

53. PCR conditions:

**Table:** PCR cycling conditions

Steps	Temperature	Time	Cycles
Initial Denaturation	95°C	5 min	1
Denaturation	95°C	1 min	15 cycles
Annealing	58°C	30 sec	
Extension	72°C	1 min	
Final extension	72°C	10 min	1
Hold	10°C	forever

***Note:*** Do not spin the beads.

### Library cleanup

**Timing: 0.5 h**

Remove excess primers using VAHTS DNA Clean Beads.

54. 30 min before PCR is completed, equilibrate VAHTS DNA Clean Beads from 4°C to room temperature.
55. Add 1× DNA Clean Beads to the PCR products, mix thoroughly by pipetting, and incubate at room temperature for 5 min.
56. Place the PCR tube on a magnetic rack, and incubate at room temperature for 5 min.
57. Remove the supernatant and wash the beads with 200 μL of fresh 80% ethanol. Incubate at room temperature for 5 min. Repeat the wash once.
58. Remove the supernatant and air-dry the beads for 2−5 min.
59. Resuspend the beads with 35 μL of 10 mM Tris-HCl (pH 7.5), and mix thoroughly by pipetting up and down 15–20 times.
60. Incubate at room temperature for 5 min to elute the DNA.
61. Place the PCR tube on a magnetic rack and incubate at room temperature for 5 min. Transfer 33 μL of the supernatant to a new PCR tube.
62. Check the concentration of PCR products with NanoDrop to determine the PCR cycles for the next step (Table 2).

**Table 2:** Recommended tagged PCR cycle numbers based on purified DNA concentration

DNA concentration (ng/μl)	Amplification cycles (*n*)
>2	8
0.5–2	10
<0.5	12–14

Purified DNA concentration after cleanup is used as a guide for selecting the number of cycles in the subsequent tagged PCR step.

**Pause point:** Purified DNA can be stored at −20°C for months.

### Tagged PCR

**Timing: 1–3 h**

Tag the purified PCR products with Illumina sequencing adapter sequences.

63. Set up tagged PCR reactions for each sample as below:

**Table:** 

Reagent	Amount (μL)
5× FastPfu buffer	20
dNTPs (2.5 mM each)	8
P5-I5 primer (10 μM)	4
P7-I7 primer (10 μM)	4
FastPfu	1
Purified nested PCR product	63

Divide each sample into two 50-μL reactions.

64. PCR conditions:

**Table:** PCR cycling conditions

Steps	Temperature	Time	Cycles
Initial Denaturation	95°C	3 min	1
Denaturation	95°C	20 sec	8–14 cycles
Annealing	60°C	30 sec	
Extension	72°C	1 min	
Final extension	72°C	5 min	1
Hold	10°C	forever

**CRITICAL:** Determine the number of cycles according to the concentration of the nested PCR product.

65. Determine the concentration of the PCR products using Qubit. The concentration should be around 20–40 ng/μL. Troubleshooting 2.

**Pause point:** PCR products can be stored at −20°C for days.

66. Size-select libraries by resolving PCR products on a 2% agarose gel, excising fragments between 300–500 bp. Recover the DNA using a gel extraction kit according to the manufacturer’s instructions. Troubleshooting 3.
67. Check the library DNA concentration with Qubit.

**Pause point:** Library can be stored at −80°C for months.

### Sequencing

**Timing: 2–5 days**

68. Sequence libraries using 150-bp paired-end reads.

***Note:*** For each bait, 1–2 million raw reads are sufficient for the following analysis. For each library, 3–5 million raw reads, with 1–1.5 Gb raw data are recommended.

### Sequence-read processing

**Timing: 5 h**

We defined “on-target” sequences as the RSS ± 40 bp regions to accurately assess V(D)J segment usage.^20^ The following code was used to compute the utilization levels from these on-target reads.

69. Trim sequencing reads. The adapter sequences (AGATCGGAAGAG) are determined by the sequencing platform and the library preparation method.

>cutadapt -e 0.1 -O 3 -m 55 -j 8 --quality-cutoff 25 -a AGATCGGAAGAG -A AGATCGGAAGAG -o cutadapt/${sample}_R1.fastq.gz -p cutadapt/${sample}_R2.fastq.gz Raw/${sample}_R1.fastq.gz Raw/${sample}_R2.fastq.gz

70. Prepare metadata for each library.

**Table:** 

Metadata information									
LIBRARY	ASSEMBLY	CHR	START	END	STRAND	MID	PRIMER	ADAPTER	DESCRIPTION
Young_HTGTS_TCR_seq_Trav_mix_rep1	mm10	chr14	52428799	52428876	+		CTCGA ATGGG TACAG TTACC TGCT	ACTCTTT CCCTAC ACGACG CTCTTCC GATCT	Trav1

**CRITICAL:** “LIBRARY” is the unique name of the library, used to name most pipeline output files. “ASSEMBLY” is the reference genome (e.g., hg19, mm10) for alignment, used to locate the reference sequence and Bowtie2 index. “CHR” is the chromosome containing the bait locus (e.g., chr15). “START” and “END” are the first and last nucleotide positions of the bait locus. “STRAND” is the nested primer orientation (‘+’ or ‘-’) (Figure 2). “MID” is the barcode sequence if present at the start of the forward read; otherwise, blank. “PRIMER” is the nested primer sequence. “ADAPTER” is the adapter sequence.

71. Specify the required genome database and Bowtie2 index locations via environment variables, then run the main HTGTS processing command. Detailed information for each junction is stored in the resulting.tlx file. Troubleshooting 4.

>source /path/to/robinmeyers/profile

>export GENOME_DB=/path/to/genomes/

>export BOWTIE2_INDEXES=/path/to/genomes/mm10/bowtie2_indexes/

>TranslocWrapper.pl meta_file.txt /path/to/trimmed_fastq/

/path/to/output_directory/ --threads 24 --pipeline-opt "--no-dedup"

72. Statistics of On-target Events for Each Gene.

>files=$(ls ∗_result.tlx|sed 's/_result.tlx//g')

>for sample in $files

 do

  grep -v 'Rname' ${sample}_result.tlx|awk

'BEGIN{FS=OFS="\t"}{if ($5==1) print $3,$6,$7,$1,$18,"+";else print $3,$6,$7,$1,$18,"-"}'|sort -k1,1 -k2,2n|bedtools intersect -a - -b /path/to/Reference/Mouse/mm10/mm10.blacklist.bed -v|grep -v 'random' |grep -v 'chrUn' > ${sample}.total.sorted.bed

  bedtools intersect -a

/path/to/TR_genes.mm10_RSS+40.bed -b ${sample}.total.sorted.bed -c > ${sample}.counts.txt

 done

***Note:****Trav3-3* and *Trav3d-3* are not distinguishable; both were maintained in this analysis, but the computed distribution of reads between the segments should not be interpreted.

73. Perform productive rearrangement analysis using the core “HTGTSrep” workflow.
  ***Note:*** To quantify TCR repertoires, we followed the core workflow of “HTGTSrep” to identify the productive and nonproductive V(D)J rearrangements, as well as to analyze the motifs and length distribution of CDR3 regions.^21^
  a. After trimming, join R1 and R2 FASTQ files and convert joined and unjoined reads to FASTA format.
    >mkdir TRBJ
    >mkdir TRBJ/${sample}
    >zcat ${sample}_R1.fastq.gz| awk '{print $1}' > TRBJ/${sample}/${sample}.R1.clean.fq
    >zcat ${sample}_R2.fastq.gz| awk '{print $1}' > TRBJ/${sample}/${sample}.R2.clean.fq
    >awk '{ if(NR % 4 == 1) {print $1} }' TRBJ/${sample}/${sample}.R1.clean.fq |sed 's/@//' |sort > TRBJ/${sample}/${sample}.R1.reads.list
    >awk '{ if(NR % 4 == 1) {print $1} }' TRBJ/${sample}/${sample}.R2.clean.fq |sed 's/@//' |sort > TRBJ/${sample}/${sample}.R2.reads.list
    >comm -12 TRBJ/${sample}/${sample}.R1.reads.list TRBJ/${sample}/${sample}.R2.reads.list > TRBJ/${sample}/${sample}.comm.reads.list
    >seqtk subseq TRBJ/${sample}/${sample}.R1.clean.fq TRBJ/${sample}/${sample}.comm.reads.list > TRBJ/${sample}/${sample}.R1.even.fq
    >seqtk subseq TRBJ/${sample}/${sample}.R2.clean.fq TRBJ/${sample}/${sample}.comm.reads.list > TRBJ/${sample}/${sample}.R2.even.fq
    >fastq-join -p 8 -m 10 TRBJ/${sample}/${sample}.R1.even.fq TRBJ/${sample}/${sample}.R2.even.fq -o TRBJ/${sample}/${sample}.unjoinR1.fq -o TRBJ/${sample}/${sample}.unjoinR2.fq -o TRBJ/${sample}/${sample}.join.fq
    >fastq_masker -q 10 -Q33 -i TRBJ/${sample}/${sample}.join.fq | fastq_quality_filter -q 10 -p 98 -Q33 | fastq_to_fasta -Q33 -n | awk '{print $1}' > TRBJ/${sample}/${sample}.join.fa
    >fastq_to_fasta -i TRBJ/${sample}/${sample}.unjoinR1.fq -Q33 -n | awk '{print $1}' > TRBJ/${sample}/${sample}.unjoinR1.fa
    >fastq_to_fasta -i TRBJ/${sample}/${sample}.unjoinR2.fq -Q33 -n | awk '{print $1}' > TRBJ/${sample}/${sample}.unjoinR2.fa
  b. Align joined and unjoined reads to the IMGT V(D)J germline database using IgBLAST.^19^ The V(D)J gene sequences were obtained from IMGT.^22^
    >igblastn -query TRBJ/${sample}/${sample}.join.fa -organism mouse -germline_db_V IMGT_ALL_TRB/TRB.V.converted.fa -germline_db_D IMGT_ALL_TRB/TRB.D.converted.fa -germline_db_J IMGT_ALL_TRB/TRB.J.converted.fa -auxiliary_data mouse_gl.aux -ig_seqtype TCR -domain_system imgt -show_translation -outfmt 19 -out TRBJ/${sample}/${sample}.joined.txt -num_clonotype 0 -num_threads 16
    >igblastn -query TRBJ/${sample}/${sample}.unjoinR1.fa -organism mouse -germline_db_V IMGT_ALL_TRB/TRB.V.converted.fa -germline_db_D IMGT_ALL_TRB/TRB.D.converted.fa -germline_db_J IMGT_ALL_TRB/TRB.J.converted.fa -auxiliary_data mouse_gl.aux -ig_seqtype TCR -domain_system imgt -show_translation -outfmt 19 -out TRBJ/${sample}/${sample}.unjoinR1.txt -num_clonotype 0 -num_threads 16
    >igblastn -query TRBJ/${sample}/${sample}.unjoinR2.fa -organism mouse -germline_db_V IMGT_ALL_TRB/TRB.V.converted.fa -germline_db_D IMGT_ALL_TRB/TRB.D.converted.fa -germline_db_J IMGT_ALL_TRB/TRB.J.converted.fa -auxiliary_data mouse_gl.aux -ig_seqtype TCR -domain_system imgt -show_translation -outfmt 19 -out TRBJ/${sample}/${sample}.unjoinR2.txt -num_clonotype 0 -num_threads 16
  c. Extract sequences unambiguously mapped to a single TRB gene.
    >awk 'BEGIN{FS=OFS="\t"}{ split($12, arr, ","); if (length(arr) == 1){ sub(/\∗.∗$/, "", arr[1]); print $0,arr[1]} else { base = arr[1]; sub(/\∗.∗$/, "", base); same =1; for (i = 2; i <= length(arr); i++) { split(arr[i], subarr, "∗"); if (subarr[1] != base) { same = 0; break } } if (same) { print $0,base}}}' TRBJ/${sample}/${sample}.unjoinR1.txt > TRBJ/${sample}/${sample}.unjoinR1.tmp1.txt
    >awk 'BEGIN{FS=OFS="\t"}{ split($12, arr, ","); if (length(arr) == 1){ sub(/\∗.∗$/, "", arr[1]); print $0,arr[1]} else { base = arr[1]; sub(/\∗.∗$/, "", base); same =1; for (i = 2; i <= length(arr); i++) { split(arr[i], subarr, "∗"); if (subarr[1] != base) { same = 0; break } } if (same) { print $0,base}}}' TRBJ/${sample}/${sample}.unjoinR2.txt > TRBJ/${sample}/${sample}.unjoinR2.tmp1.txt
    >awk 'BEGIN{FS=OFS="\t"}{ split($12, arr, ","); if (length(arr) == 1){ sub(/\∗.∗$/, "", arr[1]); print $0,arr[1]} else { base = arr[1]; sub(/\∗.∗$/, "", base); same =1; for (i = 2; i <= length(arr); i++) { split(arr[i], subarr, "∗"); if (subarr[1] != base) { same = 0; break } } if (same) { print $0,base}}}' TRBJ/${sample}/${sample}.joined.txt > TRBJ/${sample}/${sample}.joined.tmp1.txt
  d. Filter unjoined reads by requiring the top V genes in R1 and R2 to match.
    >awk -F "\t" 'NR==FNR{b[$1]=$0;next} ($1 in b){split(b[$1],arr,"\t"); if($97==arr[97]){if($14=="" && arr[14]!=""){print b[$1]} else if($14!="" && arr[14]==""){print $0} else if($14!="" && arr[14]!=""){if($55>arr[55]){print $0}else{print b[$1]}} else{print $0}}}' TRBJ/${sample}/${sample}.unjoinR2.tmp1.txt TRBJ/${sample}/${sample}.unjoinR1.tmp1.txt > TRBJ/${sample}/${sample}.unjoin.tmp2.txt
  e. Filter high-quality reads using stringent annotation, score, and length thresholds.
    >cat TRBJ/${sample}/${sample}.joined.tmp1.txt TRBJ/${sample}/${sample}.unjoin.tmp2.txt |awk 'BEGIN{FS=OFS="\t"}{if($12 != "" && $55>=150 && $64>=90 && $20>=100 && $14 != "" && (($24-$23+1)>=34)) print $0}' > TRBJ/${sample}/${sample}.pass.txt
    >awk 'BEGIN{FS=OFS="\t"} {empty=1; for(i=37;i<=54;i++) if($i!="") {empty=0; break;} if(!empty) print}' TRBJ/${sample}/${sample}.pass.txt > TRBJ/${sample}/${sample}.final.txt
  f. Identify the productive and nonproductive V(D)J rearrangements.
    >genes=("TRBV1" "TRBV2" "TRBV3" "TRBV4" "TRBV5" "TRBV8" "TRBV9" "TRBV10" "TRBV12-1" "TRBV13-1" "TRBV12-2" "TRBV13-2" "TRBV13-3" "TRBV14" "TRBV15" "TRBV16" "TRBV17" "TRBV19" "TRBV20" "TRBV21" "TRBV23" "TRBV24" "TRBV26" "TRBV29" "TRBV30" "TRBV31")
    files=$(ls ∗.final.txt|sed 's/.final.txt//g')
    >for sample in $files
     do
      for gene in "${genes[@]}"; do
       awk -F '\t' -v gene="$gene" 'BEGIN {count=0} ($97 == gene && $8=="T" && $14 ∼ /TRBJ2-1/) {count++} END {if (count == 0) count = 0; print gene "\t" count}' ${sample}/${sample}.final.txt >> ${sample}.TRBJ2-1.productive.txt
       awk -F '\t' -v gene="$gene" 'BEGIN {count=0} ($97 == gene && $8=="F" && $14 ∼ /TRBJ2-1/) {count++} END {if (count == 0) count = 0; print gene "\t" count}' ${sample}/${sample}.final.txt >> ${sample}.TRBJ2-1.non-productive.txt
       awk -F '\t' -v gene="$gene" 'BEGIN {count=0} ($97 == gene && $8=="T" && $14 ∼ /TRBJ2-7/) {count++} END {if (count == 0) count = 0; print gene "\t" count}' ${sample}/${sample}.final.txt >> ${sample}.TRBJ2-7.productive.txt
       awk -F '\t' -v gene="$gene" 'BEGIN {count=0} ($97 == gene && $8=="F" && $14 ∼ /TRBJ2-7/) {count++} END {if (count == 0) count = 0; print gene "\t" count}' ${sample}/${sample}.final.txt >> ${sample}.TRBJ2-7.non-productive.txt
       awk -F '\t' -v gene="$gene" 'BEGIN {count=0} ($97 == gene && $8=="T" && $14 ∼ /TRBJ1-1/) {count++} END {if (count == 0) count = 0; print gene "\t" count}' ${sample}/${sample}.final.txt >> ${sample}.TRBJ1-1.productive.txt
       awk -F '\t' -v gene="$gene" 'BEGIN {count=0} ($97 == gene && $8=="F" && $14 ∼ /TRBJ1-1/) {count++} END {if (count == 0) count = 0; print gene "\t" count}' ${sample}/${sample}.final.txt >> ${sample}.TRBJ1-1.non-productive.txt
      done
     done
  g. Analyze the motifs and length distributions of CDR3 regions.
    >files=$(ls ∗.CDR3.txt|sed 's/.CDR3.txt//g')
    >for sample in $files
     do
     grep -v 'Count' ${sample}.CDR3.txt | awk 'BEGIN {FS="\t"; OFS="\t"} {sum += $1; data[NR] = $0} END {for (i = 1; i <= NR; i++) {split(data[i], row, FS); printf "%s\t%.6f\n", data[i], (row[1]/sum)∗100}}' > ${sample}.CDR3.percent.txt
     done
    >files=$(ls ∗.final.txt|sed 's/.final.txt//g')
    >for sample in $files
     do
      awk 'BEGIN{FS=OFS="\t"}{if ($50 != "" && $14 ∼ /TRBJ2-1/ && length($50)==11) print $50}' ${sample}.final.txt > ${sample}.TRBJ2-1.CDR3aa11.txt
      awk 'BEGIN{FS=OFS="\t"}{if ($50 != "" && $14 ∼ /TRBJ2-7/ && length($50)==11) print $50}' ${sample}.final.txt > ${sample}.TRBJ2-7.CDR3aa11.txt
      awk 'BEGIN{FS=OFS="\t"}{if ($50 != "" && $14 ∼ /TRBJ1-1/ && length($50)==11) print $50}' ${sample}.final.txt > ${sample}.TRBJ1-1.CDR3aa11.txt
      awk 'BEGIN{FS=OFS="\t"}{if ($50 != "" && $14 ∼ /TRBJ2-1/ && length($50)==12) print $50}' ${sample}.final.txt > ${sample}.TRBJ2-1.CDR3aa12.txt
      awk 'BEGIN{FS=OFS="\t"}{if ($50 != "" && $14 ∼ /TRBJ2-7/ && length($50)==12) print $50}' ${sample}.final.txt > ${sample}.TRBJ2-7.CDR3aa12.txt
      awk 'BEGIN{FS=OFS="\t"}{if ($50 != "" && $14 ∼ /TRBJ1-1/ && length($50)==12) print $50}' ${sample}.final.txt > ${sample}.TRBJ1-1.CDR3aa12.txt
      awk 'BEGIN{FS=OFS="\t"}{if ($50 != "" && $14 ∼ /TRBJ2-1/ && length($50)==13) print $50}' ${sample}.final.txt > ${sample}.TRBJ2-1.CDR3aa13.txt
      awk 'BEGIN{FS=OFS="\t"}{if ($50 != "" && $14 ∼ /TRBJ2-7/ && length($50)==13) print $50}' ${sample}.final.txt > ${sample}.TRBJ2-7.CDR3aa13.txt
      awk 'BEGIN{FS=OFS="\t"}{if ($50 != "" && $14 ∼ /TRBJ1-1/ && length($50)==13) print $50}' ${sample}.final.txt > ${sample}.TRBJ1-1.CDR3aa13.txt
     done

## Expected outcomes

The expected outcomes of this protocol are sonicated genomic DNA fragments, sequencing-ready libraries, and annotated TCR rearrangement datasets. Sufficient sonicated fragments of genomic DNA should appear as a smear ranging from approximately 200 bp to 1.5 kb, with a peak around 750 bp (Figure 4A). After tagged PCR, the PCR products should show a broad size distribution of approximately 200–900 bp (Figure 4B). After gel purification, the final libraries should show a broad size distribution of approximately 300–500 bp. Successful HTGTS-TCR-seq should recover bait-associated *Tcra* or *Tcrb* rearrangement products and support downstream quantification of segment usage, productive and nonproductive rearrangements, and CDR3 features. Because the assay performance can vary on different cell types, TCR locus, and bait primer design, the distribution and abundance of recovered rearrangement products may differ across samples. These outputs can be used to compare TCR repertoire characteristics across distinct cell populations and biological conditions.

## Quantification and statistical analysis

**Figure 5:**
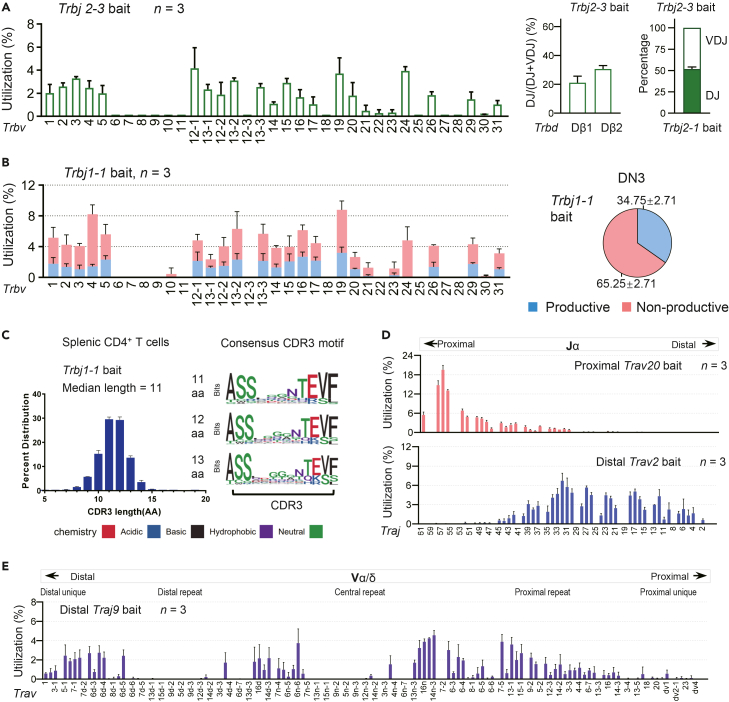
Representative analytical outputs of HTGTS-TCR-seq (A) On-target analysis using *Trbj2-3* bait, showing the usage frequency of Vβ, Dβ, and frequency of VDJ/(DJ+VDJ) rearrangement. (B) Productive rearrangement analysis using *Trbj1-1* bait, showing the frequency of each Vβ (left) and the overall distribution (right). (C) CDR3 length distribution and consensus motif analysis in splenic CD4^+^ T cells using *Trbj1-1* bait. (D) On-target analysis using *Trav20* and *Trav2* baits illustrating Jα usage frequencies. (E) On-target analysis using *Traj9* bait illustrating Vα usage frequencies. Data shown in this figure were adapted from previously published HTGTS-TCR-seq datasets, with visualization modified for presentation.^1^ Data are represented as the mean ± s.d.

To quantify the utilization level of V, D, and J segments in TCR α and β gene rearrangements, we defined “on-target” sequences as the 40-bp regions flanking the recombination signal sequences (RSS) (RSS ± 40 bp) (Figures 5A, 5D, and 5E). Reads assigned to these regions were used to calculate segment usage frequencies. The aligned number of rearrangements can be found in the statistics file. To control for differences in sample size and depth of sequencing, the utilization of each gene segment was normalized to the percent of total reads ∑(Vβ+Dβ) in Jβ bait, ∑(Vα) in Jα bait, and ∑(Jα) in Vα bait.

Productive and nonproductive rearrangements, as well as CDR3 motif usage and length distributions, were quantified from high-quality annotated reads. These reads were used to quantify CDR3 length distribution, motif usage, and the relative abundance of productive rearrangement products (Figures 5B and 5C). The same bait set, filtering criteria, and analysis parameters were applied across samples for comparative analysis.

## Limitations

This protocol has several limitations. First, genomic DNA fragmentation is required to constrain LAM-PCR product length, but sonication may disrupt some target sequences. In addition, broad capture of the highly diverse TCR repertoire requires relatively large amounts of input genomic DNA, which limits application to rare samples. Second, bait primer design is restricted by primer spacing and sequence similarity, and highly homologous or repetitive regions cannot be efficiently targeted with unique primers, thereby limiting coverage of all possible rearrangement events. Third, amplification efficiency is still influenced by template length and bait position. Although LAM-PCR reduces this bias, it does not eliminate it, and absolute quantification requires higher DNA input together with UMI-based library design and analysis. Finally, the current workflow does not distinguish chromosomal rearrangements from persistent excision circles and is optimized primarily for αβ T cells rather than comprehensive γδ T cell repertoire analysis.

## Troubleshooting

### Problem 1

Low genomic DNA yield or poor DNA quality (related to Step 19).

### Potential solution

• Use freshly prepared or properly stored samples and ensure complete cell lysis and DNA recovery.
• Check DNA concentration and purity before sonication.
• Degraded DNA or contaminated samples can reduce library yield and downstream amplification efficiency and reduce confidence in the results.

### Problem 2

Low library concentration after tagged PCR (related to Step 65).

### Potential solution

• Avoid repeated freeze-thaw cycles of the adapter by preparing aliquots before use.
• Check the storage time and handling conditions of the EasyTaq polymerase used in nested PCR and the FastPfu polymerase used in tagged PCR, as reduced enzyme activity in either step can result in weak amplification and low final library yield.
• If necessary, increase the tagged PCR by 2–3 additional cycles to improve library recovery.

### Problem 3

Tagged PCR products show a smear enriched in high-molecular-weight fragments (related to Step 66).

### Potential solution

• Genomic DNA may not have been sufficiently sonicated. Perform an additional sonication step if genomic DNA has not been sufficiently fragmented.
• Reduce the number of tagged PCR cycles in subsequent reactions. When establishing a new sample type or bait set, use a small amount of purified DNA for pilot amplification to determine the optimal cycle number before processing the full sample.

### Problem 4

Low recovery of on-target reads (related to Step 71).

### Potential solution

• Re-evaluate the bait primer design, including specificity, spacing, GC content, and annealing temperature.
• Check sequencing quality, adapter trimming, and alignment settings. Primers targeting highly homologous or repetitive regions may reduce mapping accuracy and on-target recovery.

## Resource availability

### Lead contact

Further information and requests for resources should be directed to and will be fulfilled by the lead contact, Hai-qiang Dai (haiqiang.dai@sibcb.ac.cn).

### Technical contact

Further information and questions about this protocol should be directed to the technical contact, Rui Luo (luorui@sibcb.ac.cn) and Yawei Song (songyawei2022@sibcb.ac.cn).

### Materials availability

This study did not generate unique biological materials or reagents.

### Data and code availability

No new datasets were generated for this protocol. The representative analyses shown in Figure 5 were generated by reanalyzing previously published HTGTS-TCR-seq datasets from Luo et al.^1^ The published article includes the datasets and code used in the original study.

## Acknowledgments

We thank the Dai laboratory members for their contributions to the study. This work was supported by the National Key Research and Development Program of China (2023YFA0913700), Natural Science Foundation of Shanghai (no. 25ZR1402524 to D.Y.), The Strategic Priority Research Program of the Chinese Academy of Sciences (XDB1000000), Shanghai Municipal Science and Technology Major Project (Genome Tagging Project), The International Partnership Program of Chinese Academy of Sciences (318GJHZ2022028FN), National Science Foundation of China (82271760), The Benyuan Charity Fund, Shanghai Rising-Star Program (23YF1452700), and the State Key Laboratory of Epigenetic Regulation and Intervention.

## Author contributions

H.-Q.D. conceptualized and designed the assay. R.L. and Y.S. developed HTGTS-TCR-seq and drafted the protocol. R.L., Y.S., and D.Y. prepared the figures and graphical abstract. R.L. performed the experiments. Y.S. wrote the pipeline and analyzed the data. H.-Q.D., W.W., D.Y., Y.X., and X.Q. edited and revised the protocol.

## Declaration of interests

Patent applications have been filed relating to the HTGTS-TCR-seq assay (application number in China: CN202510466824.6).
